# The Soret coefficients of the ternary system water/ethanol/triethylene glycol and its corresponding binary mixtures

**DOI:** 10.1140/epje/s10189-021-00134-6

**Published:** 2021-10-18

**Authors:** M. Schraml, H. Bataller, C. Bauer, M. M. Bou-Ali, F. Croccolo, E. Lapeira, A. Mialdun, P. Möckel, A. T. Ndjaka, V. Shevtsova, W. Köhler

**Affiliations:** 1 Physikalisches Institut, Universität Bayreuth, 95440 Bayreuth, Germany; 2 Universite de Pau et des Pays de l’Adour, E2S UPPA, CNRS, TotalEnergies, LFCR UMR5150, Anglet, France; 3 Mondragon Unibertsitatea, 20500 Arrasate-Mondragon, Spain; 4 MRC, CP 165/62, Université libre de Bruxelles (ULB), 50, Ave. F.D. Roosevelt, B-1050 Brussels, Belgium; 5 BRGM, Orléans, France; 6 Ikerbasque, Basque Foundation for Science, Bilbao, Spain

## Abstract

**Abstract:**

Thermodiffusion in ternary mixtures is considered prototypic for the Soret effect of truly multicomponent systems. We discuss ground-based measurements of the Soret coefficient along the binary borders of the Gibbs triangle of the highly polar and hydrogen bonding ternary DCMIX3-system water/ethanol/triethylene glycol. All three Soret coefficients decay with increasing concentration, irrespective of the choice of the independent component, and show a characteristic sign change as a function of temperature and/or composition. With the exception of triethylene glycol/ethanol at high temperatures, the minority component always migrates toward the cold side. All three binaries exhibit temperature-independent fixed points of the Soret coefficient. The decay of the Soret coefficient with concentration can be related to negative excess volumes of mixing. The sign changes of the Soret coefficients of the binaries allow to draw far-reaching conclusions about the signs of the Soret coefficients of the corresponding ternary mixtures. In particular, we show that at least one ternary composition must exist, where all three Soret coefficients vanish simultaneously and no steady-state separation is observable.

**Graphic abstract:**

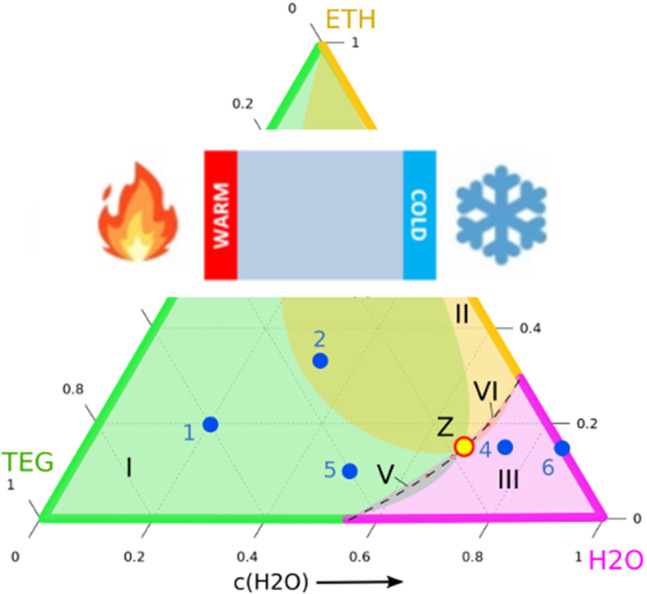

## Introduction

The Soret effect describes a thermodiffusive flow and the subsequent establishment of a composition gradient in a multicomponent fluid mixture subjected to a temperature gradient. Although most liquid mixtures of practical relevance, be it biological systems or crude oil reservoirs [1], can contain a large number of constituents, research has mainly dealt with binary mixtures. It is only recently that the focus has begun to shift to ternaries as prototypes of truly multicomponent mixtures. In the following, we will discuss ground-based measurements on the binaries of the ternary system that has been investigated during the third mission of the DCMIX microgravity project. The DCMIX project of the European Space Agency (ESA) and the Russian Space Agency (Roscosmos) has established a basis of microgravity experiments on ternary liquid mixtures subjected to a temperature gradient that can serve as convection-free references for ground experiments [2].

DCMIX consists of four individual campaigns, named DCMIX1 to DCMIX4, onboard the International Space Station (ISS). The five ternary samples of DCMIX1 were mixtures of dodecane, isobutyl benzene, and tetralin of different compositions. No undue complications were expected for this system, whose corresponding binaries were already very well characterized [3]. The focus of DCMIX2 was on mixtures of toluene, methanol, and cyclohexane, which exhibit a miscibility gap and a critical point [4]. DCMIX4 had exploratory character and contained, among additional DCMIX2-mixtures, polymer solutions, and a nanofluid [5].

The here presented work deals with the DCMIX3-system water (H2O), ethanol (ETH), and triethylene glycol (TEG) [6]. These molecules are highly polar, and the prevailing hydrogen bonding leads to much more complex interactions than the dominating dispersion interactions of the DCMIX1- and DCMIX2-mixtures. A consequence of these strong interactions is large negative excess volumes of mixing.

The aim of the following work is the investigation of diffusion and thermodiffusion along the three binary borders of the ternary Gibbs triangle of the DCMIX3 system. One of these binaries, ETH/H2O, has already been characterized in the literature [7, 8]. It shows a remarkable sign change of the Soret coefficient and is known for instabilities and oscillatory convection in double-diffusive convection experiments [9, 10]. A thorough characterization of the binaries is of great importance, as they define the values to which the transport coefficients of the ternary mixtures extrapolate in the limit of vanishing concentration of either one of the components. Since their measurement does not require complicated two-color experiments with the inversion of a potentially ill-conditioned contrast factor matrix, they can be obtained with a very good accuracy by means of, e.g., single color optical techniques. Other than for ternaries, convection can usually be avoided for binary mixtures in a Soret cell. The proper strategy is to select the direction of the temperature gradient such that the solutal separation leads to a stable stratification with the higher density at the bottom of the cell. If this requires heating from below, the stability requirement is that the thermal Rayleigh number must not exceed its critical value.

In the last part of our work, we show how the information gathered for the binaries around the perimeter of the ternary Gibbs triangle can be used to infer properties of the ternary Soret coefficients. In particular, we show that the sign changes of the Soret coefficients of the binaries lead to the existence of a singular point inside the Gibbs triangle where all three ternary Soret coefficients vanish simultaneously.

## Experimental

### Optical beam deflection

The majority of the measurements were performed by means of the well-established optical beam deflection (OBD) technique [7, 8, 11, 12]. The design of the instrument is similar to the one described in Ref. [13] with only slight modifications. The sample is inside a Soret cell with a vertical temperature gradient defined by two horizontal copper plates that are kept at a temperature difference of typically $1\,\hbox {K}$ with a stability of better than $10\,\hbox {mK}$. The lateral confinement consists of an optical glass frame and thin Teflon gaskets that together define the height of the fluid slab of $h = 1.43\,\hbox {mm}$. The path length inside the liquid is $10.00\,\hbox {mm}$. The refractive index gradient in the cell contains contributions from both the temperature and the concentration gradient, which can be separated on the basis of their very different characteristic time constants. The total refractive index gradient is read by deflection of a laser beam of $\lambda = 637\,\hbox {nm}$ at a distance of $1.325\,\hbox {m}$ behind the Soret cell, whose position is detected by a line camera.

High quality ethanol (VWR LOT 19B064011, 99.96%), triethylene glycol (Acros 99%, LOT A0389346) and de-ionized and filtrated water (resistivity $18.5\,\hbox {M}\Omega \,\hbox {cm}$, PAK-filter $0.22{\mu \,\hbox {m}}$) retrieved from a Millipore Milli-Q filtration station were used to prepare typically 3–4g of every sample to the required composition in mass fractions using an analytical balance (Sartorius BP 211 D, $\pm 0.5\,\hbox {mg}$).

Refractive indices were measured over the entire composition range for typically ten intermediate concentrations by means of an Abbe refractometer (Anton Paar, Abbemat WR-MW). The temperature dependence of the refractive index was determined interferometrically as described in Ref. [14] with the proper correction for the temperature dependence of the refractive index of the glass windows of the cell given in Ref. [8]. Based on these measurements, the refractive indices are parameterized by polynomials in the concentration *c* of the first component and the temperature $\vartheta = T - 273.15\,\hbox {K}$ in Centigrade:

1 $$\begin{aligned} n(c,T) = \begin{pmatrix} 1 &{} \vartheta \\ \end{pmatrix} \begin{pmatrix} a_{00} &{} a_{01} &{} a_{02} &{} a_{03}\\ a_{10} &{} a_{11} &{} a_{12} &{} a_{13}\\ \end{pmatrix} \begin{pmatrix} 1 \\ c \\ c^2 \\ c^3\end{pmatrix}. \end{aligned}$$

The matrix coefficients $a_{ij}$ are tabulated in Table 1. Excess volumes were computed from density measurements with an Anton Paar DSA 5000 density meter.

**Table 1:** Parameterization of the refractive indices of TEG/ETH and TEG/H2O for $\lambda = 633\,\hbox {nm}$ and for $\lambda = 532\,\hbox {nm}$ according to Eq. (1)

$a_{ij}$	Units	633 nm		532 nm	
		TEG/ETH	TEG/H2O	TEG/ETH	TEG/H2O
$a_{00}$		1.36974	1.3337	1.37223	1.33714
$a_{10}$	$10^{-4}\,{\hbox {K}^{-1}}$	$-$4.4696	$-$0.7593	$-$4.1728	$-$0.9207
$a_{01}$		0.06618	0.11976	0.07283	0.11942
$a_{11}$	$10^{-4}{\,\hbox {K}^{-1}}$	3.2519	$-$3.1494	1.3316	$-$2.1410
$a_{02}$		0.02639	0.04861	0.02124	0.05002
$a_{12}$	$10^{-4}{\,\hbox {K}^{-1}}$	$-$2.1476	$-$1.5595	$-$0.5566	$-$2.8940
$a_{03}$		–	$-$0.04041	–	$-$0.04054
$a_{13}$	$10^{-4}{\,\hbox {K}^{-1}}$	–	2.2465	–	2.6673

### Optical digital interferometry and counter-flow cell

Diffusion and Soret coefficients of selected temperatures and compositions were also measured by means of optical digital interferometry (ODI) and the diffusion coefficients at the two dilute limits of TEG/ETH with a counter-flow cell (CFC). Similar to OBD, the ODI instrument uses the Soret cell and optical diagnostics. It differs by the cell size and by the approach to the interpretation of the optical signal. The Soret cell used in the ODI setup has a square glass frame with an inner size of $18.00\times 18.00{\,\hbox {mm}^2}$. The frame is clamped between two metal plates with intermediate seals made of a special thermally conductive rubber. The total diffusion path (plate-to-plate distance) is equal to $h=6.26$ mm. This relatively large cell height limits the measurements to mixtures with a positive Soret coefficient and a corresponding stable separation. The temperature difference applied to the cell depended on the mixture under investigation. The separation in TEG/H2O-mixture was studied at ${\varDelta } T=4.00$ K, while the applied temperature difference was 6.00 K for the TEG/ETH-mixture with its smaller optical signal. The stability of the temperature regulation, estimated as the standard deviation of ${\varDelta } T$ records, is around 1 mK. The refractive index gradients appearing in the liquid inside the cell due to thermal or solutal inhomogeneities are sensed by an expanded and collimated laser beam of $\lambda =532$ nm, directed into a Mach–Zehnder interferometer, with the cell being placed in one arm of the interferometer. The optical phase variation is then extracted from the raw interference fringe patterns using a 2-D Fourier transform technique. The temporal and spatial variation of the refractive index along the diffusion path is fitted to different analytical solutions describing the Soret separation in this geometry, allowing to simultaneously extract both diffusion and Soret coefficients. More information on the instrument and the image processing can be found in Refs. [15, 16].

The isothermal diffusion at dilute limits was measured by a similar interferometer using the same data extraction approach. The counter-flow cell for the diffusion study is a metal frame with rectangular opening of $20.0 \times 5.0{\,\hbox {mm}^2}$ clamped between two optical windows using PTFE gaskets. The liquid filled space between the inner surfaces of the windows is $5.00\,\hbox {mm}$. Two inlets located at the top and the bottom of the cell allow injection of two solutions of slightly different concentrations; the heavier one is injected from the bottom to avoid instability. Two outlets located symmetrically on lateral walls at the mid-height of the cell, at $10.0\,\hbox {mm}$ ensure the formation of a sharp interface between both solutions during injection. After the injection stop and sealing of the ports, the interface elution due to diffusion is monitored in time along the diffusion path. More details of the instrument and the data extraction are available in Refs. [17, 18].

Some chemicals (TEG and ETH) used for the experiments conducted with ODI and CFC setups were equivalent by grade and manufacturer to ones used in the OBD experiments, while extra pure deionized water was purchased from Acros Organics (LOT A0396624).

### Nonequilibrium fluctuations and shadowgraphy

Additional measurements for TEG/H2O-mixtures at selected compositions and temperatures were performed by means of the dynamic shadowgraphy technique (SG), which is based on optical detection of nonequilibrium fluctuations (NEFs). These measurements are described in full detail in Ref. [19] and will only briefly be summarized. The results are included here, since they are based on somewhat different principles and very nicely align with the OBD and ODI experiments.

A fluid submitted to a gradient of temperature or concentration shows thermal and/or solutal nonequilibrium fluctuations that happen at all wavelengths, whose amplitude can be orders of magnitude larger than that of the equilibrium ones and whose size can grow up to macroscopic scales [20, 21], so that they are usually referred to as ‘giant’ fluctuations [21–23]. The associated refractive index fluctuations generate scattered beams that interfere with the transmitted one. The light intensity modulations can be collected by a pixelated sensor and analyzed in order to extract thermophysical properties of the fluid [24].

The employed shadowgraph setup is similar to the one described in Ref. [25]. A Soret cell of $25\,\hbox {mm}$ diameter contains the liquid sample that is vertically confined by two horizontal square sapphire windows at a distance of $h=2\,\hbox {mm}$. Their temperatures are regulated by two Peltier elements with a central circular aperture of $13\,\hbox {mm}$ in diameter. Contrary to OBD and ODI, the observation is not perpendicular to but rather along the direction of the temperature gradient.

Thermodiffusion experiments were performed at mean temperatures of 20, 25, and $30\,{^\circ \hbox {C}}$ with a temperature difference of $20\,\hbox {K}$ between the two sapphire windows. The temperature gradient was anti-parallel to gravity for $c=0.3$ (heating from above) and parallel for $c=0.5$ and 0.7 (heating from below). Once the steady state is achieved, a typical experiment consists of recording a series of images for a given acquisition frame rate. The analysis of each image series is performed by means of the Differential Dynamic Algorithm through a custom program taking advantage of GPU parallel execution [26, 27] in order to extract the structure function of the images [25]. The fit of a model temporal correlation function to the structure function allows to extract the decay times of the nonequilibrium fluctuations of the concentration and eventually obtain an indirect measurement of the mass diffusion and the Soret coefficients of the mixture [19, 25].

We also carried out free isothermal diffusion experiments for c = 0.5 and 0.7 using a stainless steel annulus with thickness of $h = 10.0\,\hbox {mm}$. Two inlets and two outlets allow the superimposition of two fluid layers of equal thickness and different concentration. Once the two layers are in place, the diffusion process is followed by recording series of images in time. Details of this cell and of the filling procedure are also given in Ref. [19]. The reported diffusion coefficients measured by SG are mean values from the isothermal measurements and the experiments with a temperature gradient.

## Results and discussion

### The binary borders

We report and discuss results for all three binary borders of the ternary DCMIX3 system consisting of H2O, ETH, and TEG. The results for the binaries TEG/H2O and TEG/ETH are new, the system ETH/H2O has previously been studied by Kolodner et al. [7] and in our laboratory [8]. The numerical values of the transport coefficients of ETH/H2O can be found in these two original publications and will not be repeated here. The discussion and the numerical fits will focus on the OBD-measurements, which represent a complete and internally consistent data set. The results obtained by the other experimental techniques, which are generally in good agreement but over a more limited parameter range, will be compared and discussed where appropriate.

All OBD-measurements have been evaluated following the protocol described in, e.g., Refs. [8, 28]. The experiment starts with an isothermal, homogeneous sample to which a constant temperature gradient is applied at $t=0$ by ramping the temperature of one plate up and the temperature of the opposite plate down by the same amount of typically $\delta T/2 = 0.5\,\hbox {K}$, thereby keeping the mean sample temperature constant. Assuming sufficient time scale separation, the rise time of the temperature gradient can be neglected. The formation of the concentration gradient is described on the basis of the extended diffusion equation for the mass fraction *c*(*x*, *t*) of the first component:

2 $$\begin{aligned} \frac{\partial c}{\partial t} = D \nabla ^2 c + D_T c(1-c) \nabla ^2 T. \end{aligned}$$

Both the diffusion coefficient *D* and the thermodiffusion coefficient $D_T$ are assumed constant within the 1-Kelvin-temperature variation in the cell. The Soret coefficient $S_T=D_T/D$ determines the concentration gradient in the nonequilibrium steady state. For sufficiently small Soret coefficients $S_T \ll 1/\delta T$, as prevalent in our experiments, the product $c(1-c)$ can be assumed constant. The transient beam deflection signal is fitted by an analytic solution of Eq. (2) to obtain *D* from the characteristic time constant $\tau = h^2/D$. For $D_T$ and $S_T$, the beam deflections need additionally be transformed from the refractive index to the concentration space by means of the optical contrast factors $(\partial n / \partial T)_{p,c}$ and $(\partial n / \partial c)_{p,T}$.

The focus of our discussion is on the Soret coefficients, but we also document the diffusion coefficients for reference. Our measured diffusion coefficients for TEG/H2O and TEG/ETH are tabulated as functions of concentration and temperature in Tables 2 and 3, respectively. The corresponding Soret coefficients can be found in Tables 4 and 5.

**Table 2:** Diffusion coefficients of TEG/H2O as a function of TEG-concentration *c* and temperature as obtained by OBD, SG, and ODI. SG-data from Ref. [19]

	*c*	*D* [$10^{-10}{\,\hbox {m}^2/\hbox {s}}$]						
		$10\,{^\circ \hbox {C}}$	$15\,{^\circ \hbox {C}}$	$20\,{^\circ \hbox {C}}$	$25\,{^\circ \hbox {C}}$	$30\,{^\circ \hbox {C}}$	$35\,{^\circ \hbox {C}}$	$40\,{^\circ \hbox {C}}$
OBD	0.05	4.6(.2)	5.9(.2)	5.9(.2)	7.2(.2)	7.6(.3)	8.9(.3)	
	0.3	3.4(.2)	4.1(.2)	4.7(.2)	5.2(.3)	6.2(.3)	7.0(.3)	7.8(.4)
	0.5	2.3(.1)	2.7(.1)	3.3(.2)	3.8(.2)	4.5(.2)	5.2(.3)	6.0(.3)
	0.7		1.4(.1)	1.9(.1)	2.4(.1)	2.9(.1)	3.4(.2)	4.1(.2)
	0.9		0.9(.1)	1.2(.1)	1.6(.1)	1.9(.1)	2.4(.1)	
SG	0.3			4.79(.12)	5.46(.15)	6.4(.3)		
	0.5			3.3(.1)	3.86(.14)	5.4(.3)		
	0.7			2.09(.12)	2.33(.06)	2.86(.13)		
ODI	0.05			6.2(.3)		8.0(.2)		
	0.1			6.0(.3)		7.6(.2)		
	0.15	4.1(.2)		5.6(.3)		7.2(.2)		
	0.18	4.0(.2)		5.4(.3)		6.9(.2)		
	0.2			5.3(.3)		6.0(.2)		
	0.25	3.6(.2)		4.9(.2)		6.4(.2)		8.2(.2)
	0.3			4.5(.2)		5.9(.1)		
	0.4			3.6(.2)		4.9(.1)

**Table 3:** Diffusion coefficients of TEG/ETH as a function of TEG-concentration *c* and temperature as obtained by OBD, ODI, and CFC

	*c*	*D* [$10^{-10}\,{\hbox {m}^2/\hbox {s}}$]				
		$10\,{^\circ \hbox {C}}$	$15\,{^\circ \hbox {C}}$	$20\,{^\circ \hbox {C}}$	$25\,{^\circ \hbox {C}}$	$30\,{^\circ \hbox {C}}$
OBD	0.2		4.7(.2)		4.9(.2)	5.4(.3)
	0.3	2.6(.1)	3.2(.2)	3.7(.2)	4.1(.2)	4.6(.2)
	0.5	1.6(.1)	2.0(.1)	2.5(.1)	3.1(.2)	3.4(.2)
	0.7	1.3(.1)	1.5(.1)	1.8(.1)	2.0(.1)	2.5(.1)
	0.9		0.81(.05)	0.99(.05)	1.2(.1)	1.3(.1)
ODI	0.1	3.39(.09)	4.3(.3)			
	0.15	3.1(.2)	3.9(.4)			
	0.2	3.05(.06)				
	0.3	3.0(.2)				
	0.4	2.5(.1)				
CFC	0.0015	4.9(.1)	5.54(.09)	5.88(.09)	6.5(.2)	7.0(.2)
	0.998		0.62(.03)	0.74(.04)		1.03(.09)

**Table 4:** Soret coefficient of TEG/H2O as a function of TEG-concentration *c* and temperature as obtained by OBD, SG, and ODI. SG-data from Ref. [19]

	*c*	$S_T$ [$10^{-3}\,{1/\hbox {K}}$]						
		$10\,{^\circ \hbox {C}}$	$15\,{^\circ \hbox {C}}$	$20\,{^\circ \hbox {C}}$	$25\,{^\circ \hbox {C}}$	$30\,{^\circ \hbox {C}}$	$35\,{^\circ \hbox {C}}$	$40\,{^\circ \hbox {C}}$
OBD	0.05	9.0(.5)	8.1(.4)	7.6(.4)	7.3(.3)	7.6(.3)	7.0(.3)	
	0.3	2.0(.1)	2.2(.1)	2.4(.1)	2.5(.1)	2.6(.2)	2.6(.2)	2.7(.2)
	0.5	$-$1.34(.05)	$-$1.16(.07)	$-$1.00(.06)	$-$0.85(.05)	$-$0.74(.04)	$-$0.65(.04)	$-$0.56(.03)
	0.7		$-$5.0(.3)	$-$4.5(.3)	$-$4.3(.2)	$-$4.1(.2)	$-$3.9(.2)	
	0.9		$-$7.8(.5)	$-$7.4(.4)	$-$7.0(.4)	$-$6.7(.4)	$-$6.4(.4)	
SG	0.3			2.3(.3)	2.3(.3)	2.0(.4)		
ODI	0.05			6.9(0.3)		6.7(.3)		
	0.1			6.0(.4)		6.0(.2)		
	0.15	5.0(.3)		5.0(.3)		5.1(.3)		
	0.18	4.2(.2)		4.5(.2)		4.6(.2)		
	0.2			4.1(.2)		4.3(.3)		
	0.25	2.9(.1)		3.2(.1)		3.4(.2)		3.4(.2)
	0.3			2.2(.1)		2.5(.1)		
	0.4			0.63(.06)		0.89(.04)

**Table 5:** Soret coefficient of TEG/ETH as a function of TEG-concentration *c* and temperature as obtained by OBD and ODI

	*c*	$S_T$ [$10^{-3}\,{1/\hbox {K}}$]				
		$10\,{^\circ \hbox {C}}$	$15\,{^\circ \hbox {C}}$	$20\,{^\circ \hbox {C}}$	$25\,{^\circ \hbox {C}}$	$30\,{^\circ \hbox {C}}$
OBD	0.1		0.47(.02)			
	0.2		0.36(.01)	0	$-$0.27(.01)	$-$0.56(.02)
	0.3	0.47(.02)	0.16(.01)	$-$0.14(.01)	$-$0.39(.02)	$-$0.59(.03)
	0.5	0.09(.01)	$-$0.20(.01)	$-$0.39(.02)	$-$0.57(.03)	$-$0.74(.04)
	0.7	$-$0.52(.03)	$-$0.60(.07)	$-$0.7(.1)	$-$0.8(.2)	$-$0.8(.2)
	0.9		$-$0.88(.04)	$-$0.87(.04)	$-$0.79(.03)	$-$0.87(.04)
ODI	0.1	0.92(.03)	0.55(.06)			
	0.15	0.78(.07)	0.31(.05)			
	0.2	0.61(.04)	0.26(.03)			
	0.3	0.44(.02)	0.14(.01)			
	0.4	0.191(.005)				

**Fig. 1:**
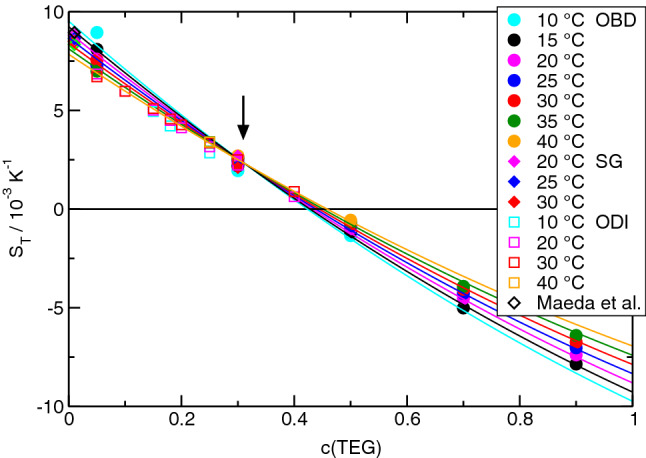
OBD-measurement (filled circles) of the Soret coefficient of TEG/H2O for different temperatures as a function of TEG-concentration *c*. The filled diamonds at $c = 0.3$ were obtained by SG and the open squares by ODI. The data at the lowest concentration of $c=0.01$ are calculated according to Maeda et al. [31] as $S_T = 9.4\times 10^{-3}\,{\hbox {K}^{-1}} - 3.0\times 10^{-5}\,{\hbox {K}^{-2}} T$ (open diamonds). The solid lines represent a simultaneous fit of Eq. (4) to all OBD-data

**Fig. 2:**
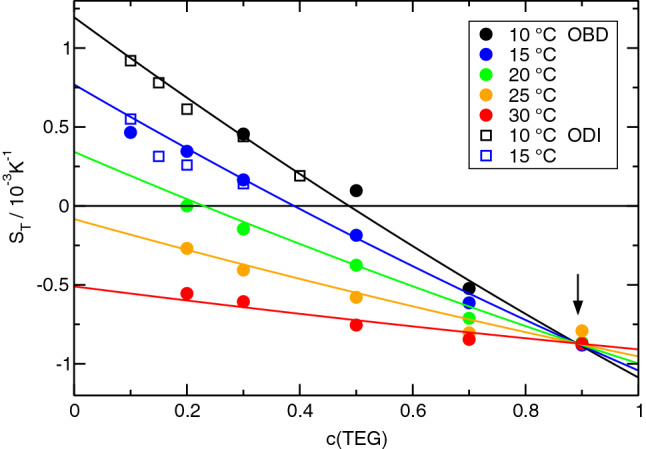
Soret coefficient of TEG/ETH for different temperatures as a function of TEG-concentration *c*

**Fig. 3:**
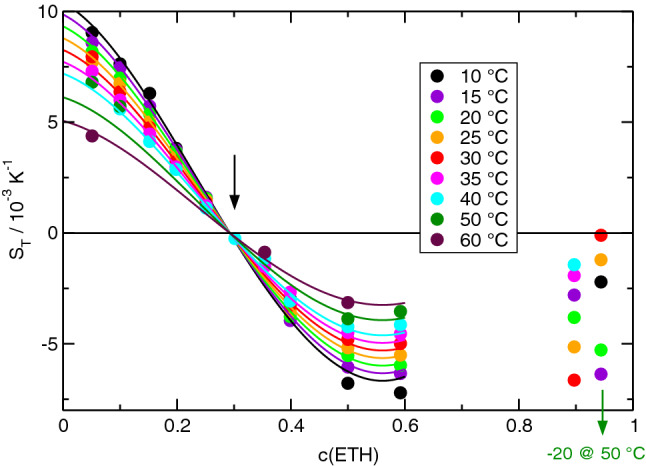
Soret coefficient of ETH/H2O for different temperatures as a function of ETH-concentration *c*. Data from Ref. [8]

The Soret coefficients of the three binaries are plotted in Figs. 1, 2, and 3. For ETH/H2O (Fig. 3), we have used only data that were measured in our own laboratory. As shown in Ref. [8], they perfectly agree with older results of Kolodner et al. [7] and also with data over a smaller parameter range by Wiegand et al. [29] and Zhang et al. [30]. Our OBD-, SG- and ODI-data for the Soret coefficient of TEG/H2O are compared with results from Ref. [31] at the lowest concentration. In the following, the prominent features that are common to all three mixtures shall be discussed.

The first obvious observation is the sign change of the Soret coefficients of all three systems as a function of concentration and/or temperature. Such sign changes, where the components invert their thermodiffusive migration direction, have been reported in the literature also for colloids [32] but they are rare for molecular systems.

The second observation is the direction of the sign change. All Soret coefficients decrease with concentration. They are positive for small and become negative for large *c*. Only for TEG/ETH at the two highest temperatures, $S_T(c)$ is always negative, but the decreasing nature of $S_T(c)$ is still preserved (Fig. 2). Keeping in mind that the given Soret coefficient is the one of the first component and that $S_T$ changes its sign when the numbering of the components is reversed, this means that the minority component always migrates to the cold side in the two dilute limits. Accordingly, the majority component goes to the hot side. It is important to understand that swapping of the components changes the sign of $S_T$ and, thus, does not change the decaying nature of the curves with positive Soret coefficients for small and negative ones for large concentrations.

The third observation relates to the temperature dependence of the Soret coefficient. For every mixture, there exists a temperature-independent fixed point of $S_T$ at a certain concentration $c_f$. In TEG/H2O, it is at $c_f \approx 0.31$ with a positive value of $S_T$, in TEG/ETH it is at $c_f \approx 0.89$ with a negative $S_T$ and in ETH/H2O it is at $c_f \approx 0.29$ with a vanishing Soret coefficient $S_T(c_f) \approx 0$. The fixed points are marked in the figures by arrows. In any case, the curves $S_T(c)$ for different temperatures pivot around the fixed point in a way such that $S_T$ decreases with increasing temperature for $c<c_f$ and increases for $c>c_f$. Together with the general decrease in $S_T$ with increasing *c*, this implies that $S_T$ approaches $S_T(c_f)$ with increasing temperature. There are also sign changes of $S_T$ as a function of temperature at certain concentrations, e.g., for TEG/ETH around $c = 0.3$ (Fig.  2). No such temperature-induced sign changes are observed for ETH/H2O, where the temperature-independent fixed point coincides with $S_T(c_f) = 0$.

A temperature-independent fixed point of the Soret coefficient has also been observed for other systems. In Ref. [33] it has been suggested to write the Soret coefficient as a composition-dependent function $\alpha (c)$ multiplied by a temperature-dependent amplitude $\beta (T)$ plus a constant offset $S_T^f$ that is identified with the Soret coefficient at the fixed point:

3 $$\begin{aligned} S_T(c,T) = \alpha (c) \beta (T) + S_T^f. \end{aligned}$$

This equation with polynomials for $\alpha (c)$ and $\beta (T)$ has already been used for the ETH/H2O-system, both with the concentration measured in mole [33] and in mass fractions [8]. Following the same idea, the Soret coefficients are fitted by

4 $$\begin{aligned} S_T(c,T) = \sum _{i=0}^4 a_i c^i ~ \left( 1 + b_1 \vartheta \right) + S_T^f. \end{aligned}$$

As in Eq. (1), $\vartheta = T - 273.15\,\hbox {K}$ is the temperature in Centigrade. The fits have been performed to the OBD-measurements, which represent a complete and experimentally consistent data set. The SG- and ODI-results are additionally plotted in Figs.  1 and 2 and are in very good agreement with the OBD-data. The fit parameters for all three systems are summarized in Table 6. As already mentioned, $b_1$ is always negative and $\beta (T)$ decreases with increasing temperature. For mixtures of benzene and cyclohexane, the Soret coefficient at the fixed point could be identified with the isotopic contribution, which is related to differences of molar mass and moment of inertia, and the term $\alpha (c) \beta (T)$ with the so-called chemical contribution [33]. Despite the similar structure of $S_T(c,T)$, such an assignment is not possible for the here considered hydrogen bonding mixtures.

**Table 6:** Fit parameters for the Soret coefficients according to Eq. (4). The values for ETH/H2O are from Ref. [8]

	TEG/H2O	TEG/ETH	ETH/H2O
$a_0$	0.00777	0.00292	0.0115
$a_1$	$-$0.0268	$-$0.00370	$-$0.0154
$a_2$	0.00604	0.000471	$-$0.1453
$a_3$	–	–	0.2378
$a_4$	–	–	$-$0.0652
$b_1$ [K$^{-1}$]	$-$0.00719	$-$0.02921	$-$0.00931
$S_T^f$ [K$^{-1}$]	0.00231	$-$0.00087	0.0

Although we cannot provide a fully quantitative description of our results, it is worth pointing out another relationship for the composition dependence of $S_T$. In Ref. [34], it is shown that the Soret coefficient can be split into contributions from the pure components, $S_T^{pur}$, and a mixing term $S_T^{mix}$. Only the latter is responsible for the concentration dependence. According to Morozov’s theory [35], it is related to the excess volume of mixing $V^E$ by

5 $$\begin{aligned} S_T^{mix} \approx C \,\frac{\partial V^E}{\partial x_1} ~, \end{aligned}$$

with $x_1$ being the mole fraction of the first component and $C < 0$ a constant that depends on the solvent compressibility.

Since $V^E$ vanishes at the two ends of the concentration axis and typically goes through an extremum in between, a positive excess volume corresponds to a situation with a negative second derivative, $\partial ^2 V^E/\partial x_1^2 < 0$. Because of the negative constant $C < 0$, this relates to the situation of a Soret coefficient that increases with *c*, and vice versa for a negative excess volumes [34]. All three mixtures show a decreasing Soret coefficient, albeit the situation is not fully clear for ETH/H2O at high ETH concentrations. A few data points above $c \sim 0.9$ might hint at an increase in $S_T$ at higher concentrations, but they show a large scatter and are very unreliable because of the vanishing solutal contrast factor $(\partial n/\partial c)_{p,T}$, which changes sign around $c=0.8$. They were already excluded from the fit in Ref. [8]. With their inclusion, the Soret coefficient would no longer decrease monotonously, but the overall picture remained unchanged: there would still be the temperature-invariant fixed point with the sign change and an overall decrease in $S_T$ from the left to the right.

**Fig. 4:**
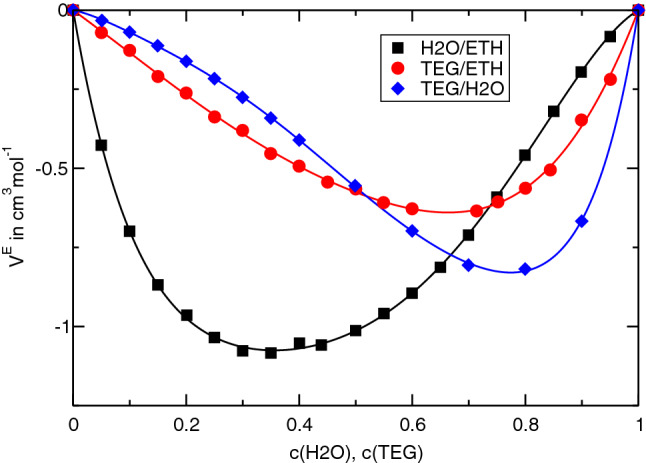
Excess volumes of mixing $V^E$ for the three mixtures TEG/H2O, TEG/ETH, and ETH/H2O at $25\,{^\circ \hbox {C}}$

Figure 4 shows the excess volumes for all three mixtures at $25\,{^\circ \hbox {C}}$ as obtained from density measurements of the pure substances and the mixtures performed with an Anton Paar DSA 5000 M densitometer. All three are negative, which is, indeed, in agreement with the observation of decreasing Soret coefficients. We want to repeat here, that a decreasing $S_T(c)$ remains decreasing under reversal of the components.

### The ternary Gibbs triangle

The knowledge of the signs of the Soret coefficients along all three binary borders allows, within certain assumptions, predictions about the signs of the Soret coefficients inside the ternary Gibbs triangle. In the following, we will use the primed Soret coefficients $S'_{T,i}$, which are the established notation for ternaries. They are defined by the concentration gradients in the Soret equilibrium, $\nabla c_i = - S'_{T,i} \nabla T$, and are related to their unprimed counterparts in the case of binary mixtures by $S'_{T,i} = c_i(1-c_i) S_{T,i}$ [36]. An in-depth discussion of frame-invariant Soret coefficients has been given by Ortiz de Zárate [37].

**Fig. 5:**
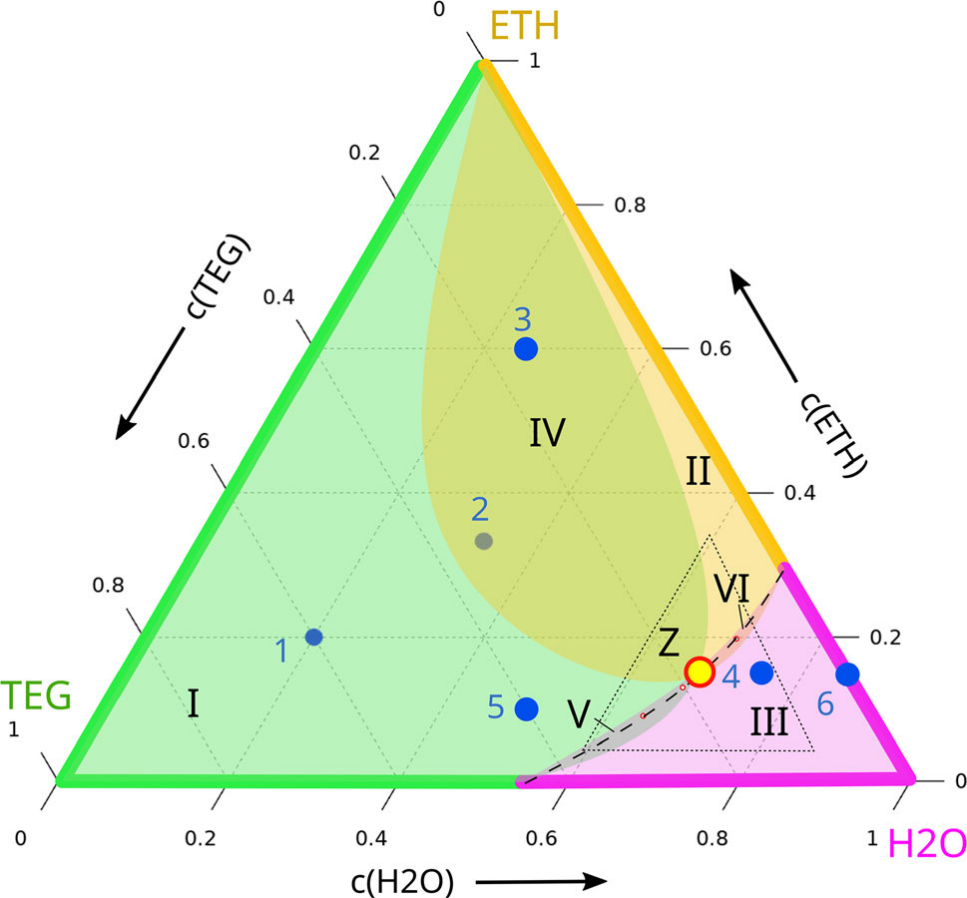
Signs of the Soret coefficients within the ternary Gibbs triangle at $25\,{^\circ \hbox {C}}$. The colored regions denote thermophilic behavior with negative Soret coefficients of the respective components. The dots 1–6 indicate the compositions of the DCMIX3 samples. Point *Z* marks the intersection of the boundaries of the three colored regions, where all three Soret coefficients vanish simultaneously. The steady-state optical signal vanishes along the dashed line (see Fig. 6). The regions I to VI are explained in the text. The triangle near the H2O corner indicates the zoom-region shown in Fig. 6

Figure 5 shows the Gibbs triangle with colors assigned to the three compounds: magenta for H2O, orange for ETH, and green for TEG. The color code along the binary borders marks the concentration range, where the respective component is thermophilic, i.e., has a negative Soret coefficient and enriches at the hot side for a mean temperature of $25\,{^\circ \hbox {C}}$. These regions are directly taken from Figs.  1, 2, and 3. The coincidence of the color code of the axes with the color of the respective component near the corners reflects our finding that, as a rule, the majority component migrates to the hot and the minority component to the cold side. The concentration of the sign change along the H2O/ETH-axis, i.e., where the color changes from magenta to orange, is temperature independent (Fig. 3) and the sign change along the TEG/H2O-axis depends only weakly on temperature (Fig. 1). The sign change concentration for TEG/ETH, on the other hand, shows a pronounced temperature dependence and shifts from $c_{TEG} \approx 0.5$ at $10\,{^\circ \hbox {C}}$ to $c_{TEG} \approx 0$ at $25\,{^\circ \hbox {C}}$, for which Fig. 5 is drawn. Very close to the selected temperature of $25\,{^\circ \hbox {C}}$, the Soret coefficient of TEG in TEG/ETH-mixtures remains negative over the entire composition range and just vanishes in the limit $c_{TEG} \approx 0$, corresponding to $c_{ETH} \approx 1$. Thus, the green color along this axis extends just up to the ETH-corner. Already at a slightly lower temperature, the green color would stop short of the ETH-corner. The following discussion does, however, not depend on these peculiar details.

Since Soret coefficients are smooth and continuous functions of the composition, and since the ternaries extrapolate to the corresponding binaries near the axes, we can draw some conclusions about the signs of the Soret coefficients of the ternaries inside the Gibbs triangle.

Let us begin with TEG. Its Soret coefficient vanishes at the ETH-corner and at the concentration of the sign change along the TEG/H2O-axis. These two points must be connected by a continuous line through the Gibbs triangle, defined as the locus where the Soret coefficient of TEG, the third component, changes sign, hence $S'_{T,3} = 0$. Together with the green sections of the axes, this line encompasses the green composition range where TEG is thermophilic. Outside, it is thermophobic. Of course, the shape of the green region inside the Gibbs triangle is only a sketch and could be more to the left or more to the right. The orange and the magenta regions can be constructed along the same rules.

From Ref. [38], it is known that both ETH (component 2) and TEG (component 3) have negative Soret coefficients at point 2, whereas the one of H2O (component 1) is positive. Hence, composition 2 must be inside both the green and the orange but outside the magenta region.

Only one negative Soret coefficient exists within regions I, II, and III. Compositions with two negative Soret coefficients define the intersection regions IV, V, and VI. Additional rules follow from mass conservation, which requires

6 $$\begin{aligned} S'_{T,1} + S'_{T,2} + S'_{T,3} = 0~. \end{aligned}$$

An immediate consequence is that the intersection of all three colors must be of size zero, since all three Soret coefficients cannot be negative at the same time. For the same reason also three positive Soret coefficients are not possible and every composition must belong to either one or two colors.

Because composition 2 is both green and orange, the boundaries of these two regions must intersect in point *Z* in a similar way as drawn in Fig. 5. Since this intersection is defined by $S'_{T,2} = S'_{T,3} = 0$, Eq. (6) immediately requires $S'_{T,1} = 0$, and the boundary of the magenta region must also pass through the intersection point *Z*.

Thus, the sign changes of the Soret coefficients of the binaries and the knowledge of their signs at one composition (point 2) inside the ternary diagram allows us to draw far-reaching conclusions about the signs of the Soret coefficients of the ternaries and to prove that at least one composition Z must exist, where all three Soret coefficients vanish simultaneously and no steady-state separation will be observed in a temperature gradient.

**Fig. 6:**
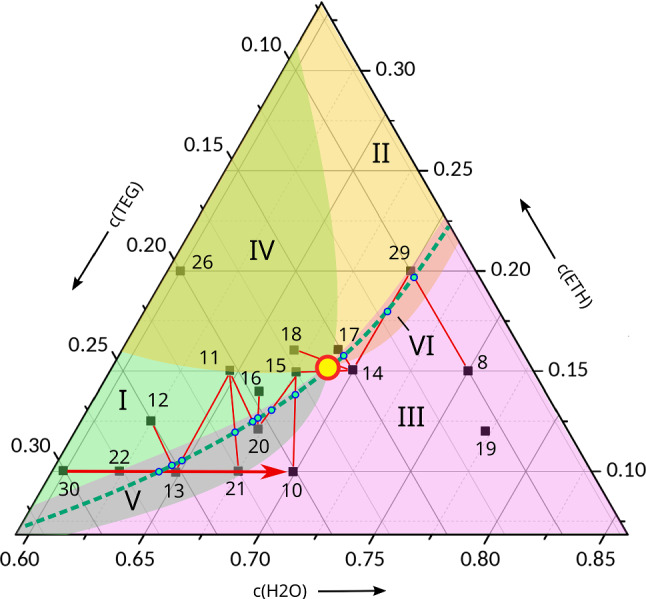
Construction of the dotted line in Fig. 5 with vanishing steady-state amplitude of the solutal OBD-signal at $25\,{^\circ \hbox {C}}$. The numbers reflect the chronological order of the measurements

**Fig. 7:**
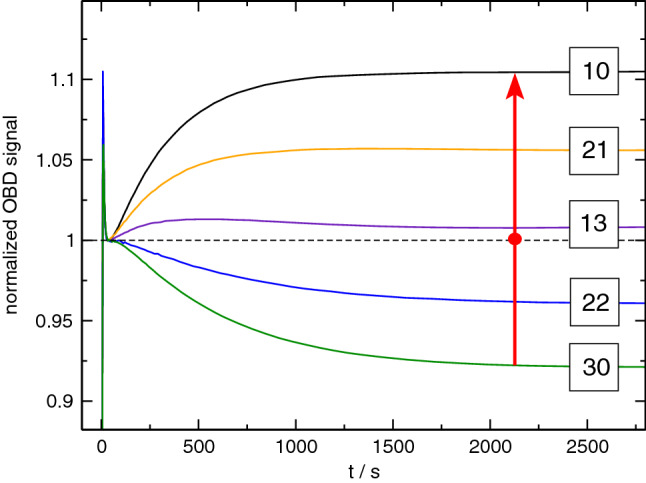
Solutal OBD-signals for measurements 30, 22, 13, 21, and 10 from Fig. 6 as indicated by red arrow. The steady-state amplitude vanishes between points 22 and 13, close to the latter

Zones V and VI in Fig. 5 are very narrow, meaning that the Soret coefficients that correspond to the two respective overlapping colors (TEG/H2O in zone V and ETH/H2O in zone VI) are very close to their sign change compositions. Since two small Soret coefficients automatically imply, according to Eq. (6), also a small Soret coefficient of the third component, the OBD-signals in zones V and VI should be very small. Since H2O has the smallest refractive index, the OBD-signal is even expected to change its sign along paths through zones I–V–III or II–VI–III.

Indeed, such a sign change within zones V and VI is observed experimentally at the dashed line in Fig. 5. This line has been constructed by measuring the OBD-signal with a single color at a large number of compositions around the expected sign change compositions. Then, pairs of compositions with different signs of their solutal OBD-amplitudes are identified. The sign change compositions are determined by linear interpolation of the two amplitudes along the connecting line in the Gibbs triangle.

Figure 6 illustrates this procedure. The composition pairs with different signs of the OBD-amplitudes are connected with thin red lines along which the compositions of asymptotically vanishing solutal OBD-signals are determined. Together with the sign changes of the Soret coefficients of the adjacent binaries, they define the dashed lines in Figs. 5 and 6.

The normalized solutal signals along the line through compositions 30-22-13-21-10 are shown in Fig. 7. The sign change occurs between compositions 22 and 13, very close to the latter. We attribute the positive amplitudes below the dashed line mainly to the negative Soret coefficient of H2O in zones III, V, and VI. The composition with vanishing signal close to composition 13 is, however, not identical to point *Z*, where all three Soret coefficients vanish. A close inspection of the solutal signal of composition 13 in Fig. 7 reveals a superposition of a fast contribution with a small positive and a slow one with a small negative amplitude.

**Table 7:** Ternary thermodiffusion and Soret coefficients of DCMIX3 sample 3 at $25\,{^\circ \hbox {C}}$. Thermodiffusion coefficients $D^\prime _{T,i}$ measured by TGC. Soret coefficients $S^\prime _{T,i}$ calculated from $D^\prime _{T,i}$ and diffusion matrix from Ref. [39]

		$D^\prime _{T,i}$ [$10^{-13}\,{\hbox {m}^2/(\hbox {sK})}$]	$S^\prime _{T,i}$ [$10^{-3}\,{1/\hbox {K}}$]
H2O	$i=1$	4.36(.13)	1.33(.08)
ETH	$i=2$	$-$3.30(.15)	$-$1.03(.09)
TEG	$i=3$	$-$1.06(.13)	$-$0.30(.07)

Thus, although the solutal steady-state optical signal asymptotically vanishes along the dashed line, it is still a superposition of two, albeit small, contributions with different signs that correspond to the two eigenmodes with different eigenvalues of the diffusion matrix. As a consequence, the vanishing asymptotic signals can still show transient amplitudes for finite times. The strict requirement for an asymptotically vanishing solutal amplitude of the optical signal reads

7 $$\begin{aligned} \left( \frac{\partial n}{\partial c_1} \right) _{p,T} S'_{T,1} + \left( \frac{\partial n}{\partial c_2} \right) _{p,T} S'_{T,2} = 0 ~. \end{aligned}$$

Because of the dispersion of the optical contrast factors, the precise position of the dashed line through regions V VI, with the exception of point *Z*, is expected to depend slightly on the employed detection wavelength. In principle, Eq. (7) could also be satisfied by a very peculiar combination of large Soret coefficients and matching optical contrast factors. Since this can be excluded at the two binary limits, and since the component separation necessarily needs to be small with changing signs within the narrow overlap regions V and VI, we consider it safe to exclude such unlikely coincidences.

Because of the vanishing solutal steady-state signal on the dashed line, it is very difficult to pin down the precise locus of point *Z*. Its position can be shifted along the dashed line and even the extreme positions at the two binary axes cannot be ruled out completely. They would still be compatible with our arguments. On the other hand, there are neither experimental observations nor theoretical arguments that would support such a very special assumption.

The sample from DCMIX3 cell number 3 with a composition of 0.25/0.6/0.15 (H2O/ETH/TEG) provides the opportunity for an additional test of the picture developed so far. It is, besides sample 2, the only other DCMIX3 sample that has a positive separation ratio and can be measured by the thermogravitational column (TGC) technique with its superior contrast factor matrix. As shown in Fig. 5, sample 3 should be in the same zone IV as sample 2 with a positive Soret coefficient for H2O and negative Soret coefficients of ETH and TEG. In order to test this prediction, TGC measurements were performed following the identical protocol as employed for sample 2 in Ref. [38]. The results are listed in Table 7. The experiments yield directly the thermodiffusion coefficients $D^\prime _{T,i}$. The Soret coefficients $S^\prime _{T,i}$ are calculated as described in Ref. [38] using the diffusion matrix from Ref. [39]. As can be seen from Table 7, the signs of the three ternary Soret coefficients are in agreement with our model.

## Summary and conclusion

We have presented Soret- and diffusion coefficients of the three binary subsystems of the ternary DCMIX3 system consisting of H2O, ETH, and TEG. All three binaries are strongly interacting hydrogen bonding mixtures with pronounced excess volumes of mixing [40]. We are not able to provide a fully quantitative model. There are, however, several remarkable properties that are common to all three mixtures but not necessarily found to the same extent in nonpolar organic liquids.

The most remarkable observation is a sign change of $S_T$ as a function of concentration—in the case of TEG/ETH only below room temperature. In all binaries, the Soret coefficient is a decreasing function, which leads, together with the sign change, to a migration of the minority components to the cold side in the dilute limits. Correspondingly, the majority component has a negative Soret coefficient and goes to the hot side. As already observed for organic liquids, all three mixtures show a temperature invariant fixed point of $S_T$ at a certain concentration, and $S_T$ can be factorized into a concentration-dependent function $\alpha (c)$ with a temperature-dependent amplitude $\beta (T)$ plus the constant offset of the fixed point (Eq. (3)).

Although these observations can qualitatively be interpreted in terms of concepts discussed in the literature for organic mixtures, a fully quantitative description is still lacking for these hydrogen bonding systems. An example is the decrease in $S_T$ with concentration, which is in agreement with predictions by Morozov [34, 35] for systems with negative excess volumes. Despite the nice qualitative agreement, we are not able to provide a quantitative model. Another example is the mentioned fixed point of the Soret coefficient and the parameterization of $S_T$ according to Eq. (3). While these terms could be identified with the isotopic and the chemical contribution to the Soret coefficient in the case of organic mixtures, such an interpretation fails for the here considered strongly interacting polar mixtures.

Based on the knowledge of the signs of the Soret coefficients for the binaries and for the symmetric ternary mixture (DCMIX3-composition 2), it is possible to reconstruct the signs and sign changes also for the ternaries within the Gibbs triangle. Since the three Soret coefficients are not independent, two small Soret coefficients automatically imply that also the third one needs to be small. This, in combination with the low refractive index of water, leads to vanishing optical signals along the dashed line through the narrow overlap regions V and VI in Fig. 5, which are close to the loci where the Soret coefficients of TEG and H2O or ETH and H2O change their signs. Though its precise location has not been nailed down, these considerations necessarily imply the existence of a certain composition, point *Z*, where all three Soret coefficients vanish simultaneously and where no separation will occur in a temperature gradient.

On purpose, we have used as little experimental input as possible for the ternary mixtures inside the Gibbs triangle. The determination of the Soret coefficients for the ternaries requires two-color experiments, as employed for the DCMIX project, and the inversion of the optical contrast factor matrix. Due to the large condition numbers of the latter, the errors introduced by this procedure are unavoidably large. An exception is composition 2, which has been investigated in Ref. [38] both under microgravity and ground conditions. In particular, the employed thermogravitational column technique provides, for this particular system, superior contrast factor matrices. But since this method only works for positive separation ratios, it is limited to the DCMIX3-mixtures 2 and 3. These problems do not exist for the binaries and the reported data are generally of a high quality. Thus, it was our goal to investigate, how much information about the ternaries can be extracted for this particular system from the knowledge of the binaries alone. Even without DCMIX3 sample 2, the conclusions would be very similar and only the orange region with the negative Soret coefficient of ETH might be drawn somewhat less toward the TEG corner. It should be remembered that the measurements shown in Fig. 6 to determine the dashed line were only made with a single color and did not involve the inversion of a contrast factor matrix.

## Data Availability

Data are available upon reasonable request from the authors.
